# The Protective Role of Physical Fitness Level Against Obesity and Body Dissatisfaction in French-Canadian Youth

**DOI:** 10.3390/jfmk10010046

**Published:** 2025-01-26

**Authors:** Mario Leone, Isabelle Thibault, Hung Tien Bui, Emilia Kalinova, Jean Lemoyne, Dominic Gagnon, Georges Larivière, Maxime Allisse

**Affiliations:** 1 Faculté de Médecine et des Sciences de la Santé, Université de Sherbrooke, Sherbrooke, QC J1H 5N4, Canada; hung.tien.bui@usherbrooke.ca; 2 Faculté d’Éducation, Université de Sherbrooke, Sherbrooke, QC J1K 2R1, Canada; isabelle.thibault@usherbrooke.ca; 3 Faculté des Sciences, Université du Québec à Montréal, Montréal, QC H2X 1Y4, Canada; kalinova.emilia@uqam.ca; 4 Département des Sciences de l’Activité Physique, Université du Québec à Trois-Rivières, Trois-Rivières, QC G8Z 4M3, Canada; jean.lemoyne@uqtr.ca; 5 Jonquière Médic, Saguenay, QC G7X 7W6, Canada; dominic.gagnon.med@ssss.gouv.qc.ca; 6 École de Kinésiologie et des Sciences de l’Activité Physique, Université de Montréal, Montréal, QC H3T 1J4, Canada; georgeslariviere111@sympatico.ca; 7 Faculté des Sciences de l’Activité Physique, Université de Sherbrooke, Sherbrooke, QC J1K 2R1, Canada; maxime.allisse@usherbrooke.ca

**Keywords:** adolescents, physical performance level, body image, anthropometric measures, socioeconomic status

## Abstract

**Background**: The obesity epidemic among adolescents significantly impacts not only their physical health but also various psychological factors, including their perception of body image. Thus, this study pursued three main objectives: (1) to update the reference standard values for all the physical fitness tests performed; (2) to examine the impact of overweight and obesity on factors influencing physical fitness in adolescents; and (3) to determine the relationship between the physical fitness level and the body image dissatisfaction among a population of French-Canadian adolescents. **Methods**: A total of 1862 adolescents aged 12 to 17 (1008 boys and 854 girls) participated in this study. Data were collected from 12 French-language high-schools from different socioeconomic backgrounds and spread across four regions of the province of Québec, Canada. Anthropometric measures (body mass, body height, body mass index (BMI), waist circumference, waist-to-height ratio) and fitness tests (aerobic power, anaerobic power, muscle endurance, muscular power, flexibility) were conducted. To assess adolescents’ body perception, a silhouette scale was used. **Results**: Standardized normative values were established for each fitness test (Lambda Mu Sigma; LMS method). In boys, performance generally improved with age, except for the V-test and sit-ups, which remained stable, and VO_2_peak, which declined during adolescence in both genders (unpaired *t*-test and Cohen’s d effect size). In girls, only the vertical jump and 30 m sprint improved with age, while the other tests stabilized by age 13. Fitness level was significantly influenced by obesity status. Boys and girls with a normal BMI performed better than those who were overweight or obese (ANOVA = *p* < 0.001 and effect size *F*). Girls appeared to be less affected by obesity status, with differences between overweight and obese groups rarely being significant (*p* > 0.05). Fitness level was also linked to body satisfaction, with satisfied adolescents generally achieving better scores than dissatisfied ones, even among those with a typical BMI. Socioeconomic status did not impact body image perception in boys (*p* = 0.351). In contrast, girls from lower socioeconomic backgrounds exhibited significantly more negative perceptions (*p* = 0.002) than their peers from more affluent families. **Conclusions**: Obesity status is strongly associated with poorer performance on fitness tests. Conversely, higher levels of physical fitness are linked to improved body image satisfaction. This positive relationship between fitness and body image holds true even for individuals with a healthy body weight (typical BMI).

## 1. Introduction

Adolescents’ physical fitness is a cornerstone of their long-term health, directly impacting their risk of developing chronic diseases later in life. Extensive research has demonstrated that a robust level of physical fitness, including both cardiorespiratory and muscular fitness, substantially decreases the likelihood of developing metabolic, physiological, psychological, and psychosocial issues [1,2,3,4]. Physical fitness is also a correlate of psychological well-being, positive self-esteem, and positive body image [5].

The childhood obesity epidemic, a global issue to which Canada is not immune, is a serious cause of concern [6,7,8,9]. While the etiology of adolescent obesity is multifaceted, encompassing a wide range of lifestyle factors, including dietary habits, certain other factors exert a significant influence and remain predominant. Thus, the surge in overweight and obesity rates, coupled with a dramatic decline in physical activity levels, seems to be closely linked to a decrease in cardiorespiratory fitness among adolescents [10,11,12]. Indeed, two recent studies conducted in Québec in 2023, based on a representative sample, provided valuable insights into the prevalence of obesity, its progression over the past 40 years, and its impact on cardiorespiratory fitness [6,10]. The findings highlight a significant decline in VO_2_peak since 1982, a trend that mirrors the sharp rise in overweight and obesity rates, which have more than tripled among adolescents in Québec over this period. It is worth noting, however, that this phenomenon is not unique to Québec and has also been observed worldwide [13,14,15].

While the relationship between obesity and cardiorespiratory fitness is well established, other physical fitness components such as muscular factors (strength, power, and endurance), flexibility, and anaerobic capacity have been the subject of less in-depth research. Indeed, deficits in fitness components beyond cardiorespiratory capacity can have negative health consequences for adolescents, including a higher risk of developing chronic conditions, both in the short term and later in life [16,17]. Although recent, rigorously conducted studies have shed more light on the global physical fitness condition of young Canadians [18,19], the impact of overweight and obesity on this condition remains a crucial issue that warrants further investigation. Given the cultural, social, and linguistic differences unique to Québec, it is preferable to develop a tailored profile of this population to identify the most effective strategies for addressing this phenomenon. A recent study conducted in 2017 by Tomkinson et al. [11] highlighted significant international variations in the trends of cardiorespiratory fitness, complicating its universal applicability. Similarly, Canada, a vast country by land area, is not a homogeneous territory and exhibits notable regional disparities. For instance, Leone et al. (2023) reported substantial interprovincial differences in obesity prevalence, ranging from 12.8% in British Columbia to double that rate in Newfoundland [6]. In this context, it is warranted to generate region-specific data that consider local particularities, even though national surveys may also be valuable in certain specific contexts.

This decline not only increases the risk of developing health issues early in life but can also interfere with various daily activities, including the ability to perform effectively in a job during adulthood. In this regard, a recent longitudinal study published by Laakso and his team in 2024 found that low cardiorespiratory fitness during adolescence was linked to diminished work capacity and decreased productivity among mid- and late-career workers [13].

Even though there is an undeniable link between the concepts of physical activity and physical fitness, some distinctions must still be taken into consideration. Physical fitness is the set of physiological, anthropometric, and functional qualities developed by the body in response to physical exercise. It is defined by optimal levels of cardiovascular capacity, strength, power, muscular endurance, and flexibility and a balanced body composition [19,20]. These attributes are essential for physical performance and overall health and well-being [21,22]. Thus, physical fitness reflects the optimal state of an individual’s physiological and physical characteristics at a given time, while the level of physical activity (or inactivity) refers to the volume of exercise performed or capable of being performed, which in turn contributes to its improvement.

If physical activity serves as a catalyst for physical fitness, the latter represents the tangible outcome of the physiological adaptations induced by this activity. It is crucial to recognize that physical fitness is a dynamic state, susceptible to fluctuations influenced by various factors, including age, gender, training intensity, and genetic predisposition. For the purpose of this study, the focus was solely on the level of physical fitness, as this aspect has been less extensively investigated, especially in relation to its associations with socio-demographic and psychosocial variables. By delving into these specific areas, we aimed to contribute to a more comprehensive understanding of the multifaceted nature of physical fitness and its implications for overall well-being.

Body image, as conceptualized by experts in the field, is an individual’s subjective perception of one’s own body, integrating a complex set of representations, evaluations, and affects [23]. It encompasses perceptual dimensions (how one sees oneself), attitudinal dimensions (judgments made about one’s appearance), and emotional dimensions (feelings associated with this image). Body image exists on a spectrum, ranging from satisfaction to deep dissatisfaction. A positive body image is more than mere acceptance of one’s physical appearance; it encompasses a holistic appreciation of one’s body, its functions, and its capabilities. It is a harmonious relationship with oneself, rooted in self-respect and self-esteem. Conversely, body image dissatisfaction is characterized by persistent discontent with one’s physical appearance, often accompanied by recurring negative thoughts and unpleasant emotions like shame or disgust [23]. This discomfort arises from a perceived discrepancy between one’s own body and societal beauty ideals, which often impose unrealistic and unattainable standards. The latter is based on the internalization of societal ideals relating to physical appearance. This notion is a major issue, as it exerts a significant influence on mental health and well-being, particularly by shaping self-esteem and body-related behaviors [24].

The substantial increase in sedentary behavior among adolescents in recent decades has underscored a strong link with the decline in their physical fitness. This shift, marked by a decrease in physical activity in favor of a more sedentary lifestyle, has significant repercussions on a teenager’s health. Sedentary behavior is not only associated with a decline in physical fitness but also impacts various psychosocial and behavioral dimensions. Among the most direct consequences, we see an alarming rise in cases of overweight and obesity within this population [4,6,7,8,9]. Moreover, it is well recognized that obesity and overweight are closely linked to higher levels of body image dissatisfaction among adolescents [25,26,27]. Conversely, numerous studies have shown that regular physical activity is associated with improved body image, notably by fostering better self-esteem and a more positive perception of physical appearance [28,29,30].

While this finding is crucial, it remains incomplete. Though physical activities or sports practice is generally beneficial, the intensity, frequency, duration, and nature of these activities play a decisive role in enhancing physical fitness and, by extension, in their effect on body image. In other words, occasional physical activity alone is insufficient to yield significant results. Regular individual ability-tailored exercise is necessary to fully harness the positive effects on physical and mental health. However, standardizing the many markers involved can be challenging. This is why using physical fitness levels measured through a battery of standardized physical tests serves as a useful criterion. Although some studies have investigated this issue [31,32], they remain few, and, to our knowledge, none focused specifically on the Canadian adolescent population. Conducting further research is therefore essential to better understand the mechanisms underlying this complex relationship and to identify the factors moderating the association between physical fitness and body image.

Thus, this study pursued three main objectives: (1) to update the reference standard values for all the physical fitness tests used; (2) to examine the impact of overweight and obesity on factors influencing physical fitness in Canadian adolescents from Québec; and (3) to determine the relationship between the level of physical fitness and body image dissatisfaction.

## 2. Materials and Methods

### 2.1. Design

This research was based on a large-scale cross-sectional epidemiological study conducted among a representative sample of adolescents aged 12 to 17 years, enrolled in schools in Québec (Canada). The data, collected between 2014 and 2017 as part of a regional survey, provide an overview of the situation prior to the COVID-19 pandemic. The use of the free versions of Grammarly and ChatGPT 3.0 was limited to detecting and correcting spelling, syntax, and grammatical errors.

### 2.2. Participants

A total of 1862 adolescents (1008 boys and 854 girls) aged 12 to 17 (boys, 14.8 ± 1.5 years; girls, 14.6 ± 1.6 years) participated in this research. Given the structure of the Québec school system, which provides for five years of high-school studies, we targeted this age range in order to include the majority of teenage students. To ensure geographic and demographic representativeness, 12 French-language high-schools were randomly selected from four Québec cities with contrasting population densities: the metropolitan areas of Montréal and Laval and the mid-sized cities of Trois-Rivières and Saguenay. This stratified sampling approach accounted for Québec’s sociodemographic diversity, characterized by a predominantly French-speaking majority, a significant allophone community concentrated primarily in the greater Montréal area, and an immigrant population comprising approximately 25% of the total population. To obtain a representative sample of urban adolescents in Québec, who constitute 85% of the total population, a proportional distribution of students was carried out based on the population of each city. The schools were randomly selected from a pool of over 145 educational institutions that had expressed interest in participating in this study. A deliberate effort was made to select schools across the socioeconomic spectrum, from affluent to highly disadvantaged, to ensure adequate representation within our sample. Schools and classes were randomly assigned. With few exceptions, all students in a given class participated in the assessment, ensuring the absence of selection bias.

A three-stage sampling strategy was shown to be suitable for choosing a representative sample of school boards, schools, and classrooms. All participating adolescents were fluent in French, allowing for all instructions, documents, and questionnaires to be administered in French, the official language of Québec. G*Power software (version 3.1.9.4) was employed to conduct a power analysis using Cohen’s d effect size to determine the necessary sample size for this investigation. A power of 0.95 and a significance level of 0.05 were set to detect small effects (d < 0.1). Based on these markers, a sample size of 1564 participants was determined to be necessary. Students and parents were informed about the study and given the option to decline participation. Additionally, each participant was free to withdraw at any time or to refuse certain tests without any penalty or prejudice. The Institutional Ethical Committee Board approved the project (no: 602-225-01). A more detailed methodological description is provided in recent publications by our group, as the current data are a subset of a larger study [6,10,33].

### 2.3. Anthropometric Measures

All selected anthropometric procedures adhered to established, recognized protocols for reliability and validity. Anthropometric variables of body mass (BM), body height (BH), body mass index (BMI), waist circumference (WC), and waist-to-height ratio (WHtR) were measured during physical education classes, one student at a time, out of sight, using methods recommended by Lohman and colleagues [34]. Kinesiology interns conducted these measurements in a separate room adjacent to the gym. BM was recorded to the nearest 0.1 kg using a Detecto scale (Webb City, MO, USA), while BH and WC was measured to the nearest 0.1 cm using a SECA model 213 stadiometer (Hamburg, Germany) and WC using a Gulick anthropometric retractable tape (Wilmington, NC, USA). According to the classification proposed by Cole et al. [35], the BMI was determined using the formula BM (kg)/BH^2^ (cm) and was categorized as normal (typical BMI), overweight, or obese. The formula WC (cm)/BH (cm) was used to determine the WHtR.

### 2.4. Physical Fitness Tests

**Figure 1:**
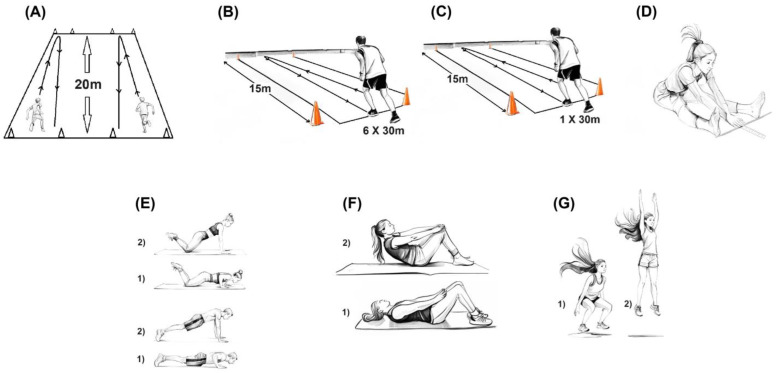
Composition of the fitness assessment test battery: (**A**) VO_2_peak; (**B**) maximal anaerobic capacity; (**C**) 30 m back-and-forth sprint; (**D**) flexibility; (**E**) push-ups; (**F**) sit-ups; (**G**) countermovement jump.

The test battery used was designed to assess the key components of physical fitness, namely, maximal aerobic power (VO_2_peak), maximal anaerobic power (6 × 30 m sprint), muscular endurance (push-ups and sit-ups), muscular power (countermovement jump and 30 m sprint), and flexibility (V-test), as shown in Figure 1. All physical tests used in this study are recognized for their validity and reliability and have been administered repeatedly to age- and sex-matched populations, in accordance with the recommendations of the assessment protocols described below. All assessments, including anthropometry, physical fitness, and body image evaluations, were conducted indoors between 9:00 AM and 3:00 PM, Monday through Friday, from October to May. Certified kinesiology interns, who had undergone 45 h of specialized training in anthropometric and physical assessment, meticulously performed all measurements. The process was closely supervised by university researchers directly involved in the project. To minimize performance impairment due to fatigue, the tests were distributed over two days. Prior to each physical testing session, a tailored 10 min warm-up was provided, designed to simulate the upcoming activities, including sprints, jumps, push-ups, abdominal exercises, and stretching.

(A) VO_2_Peak

VO_2_peak was evaluated using the 20 m shuttle run test (validity, r = 0.710; test-retest reliability, r = 0.890), a procedure that estimates the participant’s maximal aerobic power [36]. The first step involves drawing two parallel lines on the ground, 20 m apart. Participants line up, side by side, behind the starting line, with one meter of distance between each individual.

The goal of the test is to complete as many round trips as possible. The running pace is indicated by a CD emitting sound signals. Each participant must synchronize their running speed to reach the next line precisely when the sound signal is emitted, come to a complete stop, and then start back towards the starting line. The speed gradually increases every minute. The test ends when the participant is no longer able to maintain the required pace. At this point, the number of the last completed stage is recorded (each stage number is announced during the test).

(B) Anaerobic test

Adapted from the Running-Based Anaerobic Sprint Test (RAST), the aim of this test is to assess maximal anaerobic power (validity, r = 0.530; test-retest reliability, r = 0.88) [37]. It involves performing 6 maximal sprints of 30 m (15 m outward, an abrupt stop, then returning for 15 m), with 10 s rest periods between each 30 m. The time for each 30 m sprint was recorded with an accuracy of 0.01 s. The final result was calculated by summing the times of all six round trips, excluding the 10 s rest intervals.

(C) 30 m sprint test

The 30 m sprint test is designed to evaluate an individual’s muscular power output in the horizontal plane (test-retest reliability present study, r = 0.921). The outcome was determined through the RAST test (see anaerobic test procedure), where the fastest back-and-forth sprint (typically one of the initial two sprints) was chosen. Since most high-school gymnasiums in Canada lack sufficient length, a 15 m sprint with a sudden stop and return to the starting line was implemented to work within these limitations, making this test feasible in all schools. Results were recorded in seconds, with a precision of 0.01 s.

(D) V-test

The V-test, commonly referred to as the sit-and-reach test, evaluates the flexibility of the lower back, hips, and hamstring muscles (test-retest reliability, r = 0.98) [38]. The participant sits on the floor with legs extended and separated by approximately 30 cm, toes pointing upwards. The longitudinal line should be positioned between the legs, with the heels just behind the transverse line. The participant needs to be positioned at the precise intersection of both lines (zero point), ensuring a straight back and unbent knees. Hands are placed one on top of the other, palms facing down. At the signal, the participant slides their hands along the longitudinal line as far as possible, maintaining a straight back and extended legs, performing the movement smoothly without jerking. The movement stops when the limit of flexibility is reached. Distance traveled by the hands from the zero point was measured in centimeters (cm) and recorded with a precision of 0.5 cm. Each participant received two attempts, and the best result was recorded. To prevent negative values (participants failing to reach the lateral line where heels are placed), 35 cm was added uniformly to the final score of all participants [20].

(E) Cadence push-ups

As suggested by Léger and Leone [39], this test aims to evaluate the muscular endurance of the upper limb (test-retest reliability, r = 0.982). The individual lies on their stomach with hands placed on the ground at shoulder-width apart. The task involves lifting the body while maintaining support on the hands and toes for boys and on the knees for girls. The goal is to perform as many arm flexions and extensions as possible, following the prescribed rhythm of 50 repetitions per minute. The test ended when the participant could no longer maintain the prescribed rhythm or the quality of the movements deteriorated significantly.

(F) Cadence sit-ups

Adapted from the procedure suggested by Léger and Leone [39], this test aims to evaluate the endurance of the abdominal muscles (test-retest reliability, r = 0.990). The individual lies on their back, with hands placed on their thighs and knees bent and slightly apart. At the signal, the participant performs a series of controlled trunk flexions forward, sliding their hands along their thighs until reaching the top of the knees, then returns to the initial position with their back flat on the ground. The hands should remain on the thighs throughout the movement. The objective was to perform as many half sit-ups as possible, following a predetermined pace of 40 repetitions per minute. The test ended when the participant could no longer maintain the prescribed rhythm or the quality of the movements deteriorated significantly.

(G) Vertical jump

Based on the procedure suggested by Bui et al. [40], the vertical jump test (countermovement jump) assesses the ability to generate maximum explosive force in the lower limbs (validity, r = 0.944; test-retest reliability, r = 0.982). The participant stood upright between two photoelectric cells positioned on the ground. When ready, the participant jumped as high as possible utilizing an explosive extension of the legs and hips (angle between the thighs and the calves, which was approximately 135°). During the jump, arms were forcefully swung upwards to maximize momentum. The participant must land on their feet in the same starting position. The jump height was measured by the photoelectric cells and recorded in centimeters (cm). Three attempts were allowed, and the highest jump was considered for the final result. Since this method is based on the time of flight, the calculation of the maximum height was compiled from the classic formula of mechanical physics: Hmax = a × t^2^_of_/8 where Hmax is the height of the jump, t^2^_of_ is the time of flight, and a is the gravitational acceleration (−9.8 m/s^2^).

### 2.5. Assessment of Body Image

To assess adolescents’ body perception and body image, a silhouette scale was used. The Contour Drawing Rating Scale (CDRS), published in 1995 by Thompson and Gray, is widely recognized as a valid and reliable tool for assessing body image perception with a convergent validity of r > 0.70, a reliability ranging from r = 0.80 to 0.90, and an inter-rater reliability of 0.85 [41]. This scale includes two sets of nine drawings representing different body shapes ranging from very thin to obese. First, participants selected the sex-appropriate silhouette that most closely matched their current perception of their body. Then, they chose the ideal silhouette: the one they would like to have. The difference between these two choices made it possible to measure the level of body dissatisfaction, a negative gap indicating a desire to be thinner and a positive gap indicating a desire for a larger body shape. Furthermore, to enhance the validity of the body image self-assessments, an independent evaluation was conducted by kinesiology trainees for each participant. This dual-assessment approach allowed for a comparison between subjective and objective measures of body shape perceptions.

### 2.6. Statistical Analysis

Results are presented as means ± standard deviation, with 95% confidence intervals. Cohen’s effect sizes (two groups) or effect size *F* (for 3 groups) were calculated to assess the clinical importance of differences between groups. Normality of distributions was analyzed using the Shapiro–Wilk test. For variables not following a normal distribution, a Box–Cox transformation was applied. Curves were established using the Box–Cox method with cubic splines, according to the WHO recommendations [42]. Outliers were identified using the Hoaglin and Iglewicz method [43], while percentiles were calculated using the Lambda Mu Sigma (LMS) method. Agreement between self-ratings and external assessments of body image was assessed using Spearman’s correlation coefficients and Kendall’s Tau-B Comparisons between adolescents satisfied and dissatisfied with their body image were made using independent-samples Student’s t-tests, and comparisons by obesity profile (i.e., typical, overweight, or obese) were made using one-way analysis of variance. All analyses were performed using IBM SPSS version 26 software, with a significance level set at *p* < 0.05.

## 3. Results

Table 1 presents age-related changes in anthropometric and physical fitness measures. Across both sexes, most anthropometric variables showed a significant increase with age, except for the WHtR, which remained relatively stable throughout adolescence. In boys, performance in the 20 m shuttle run test as indicated by the number of completed stages, push-ups, maximal anaerobic power, 30 m sprint, and vertical jump generally improved with age. Conversely, performance in the sit-ups and the V-test remained relatively stable, while maximal oxygen consumption (VO_2_peak) decreased significantly with age.

The results observed in girls revealed a distinct developmental profile from that of boys. Specifically, only the vertical jump and 30 m sprint performances showed improvement with age. From age 13, physical performance stabilized in tests of maximal anaerobic power, the number of stages reached in the 20 m shuttle run, push-ups, and the V-test. In contrast, VO_2_peak decreased rapidly and significantly throughout adolescence, while performance in sit-ups showed a more pronounced decline from age 17 onwards. In fitness assessments, the boys generally performed better than the girls, with the exception of the range of motion test (V-test), in which the girls demonstrated greater flexibility.

Table 2, Table 3, Table 4 and Table 5 provide a comprehensive overview of percentile values for each fitness test, categorized by age and sex. These data, calculated using the LMS method and following WHO recommendations [40], cover a broad range of percentiles, from the 3rd to the 97th percentiles. The LMS values are also provided, enabling the calculation of customized percentiles as needed.

**Figure 2:**
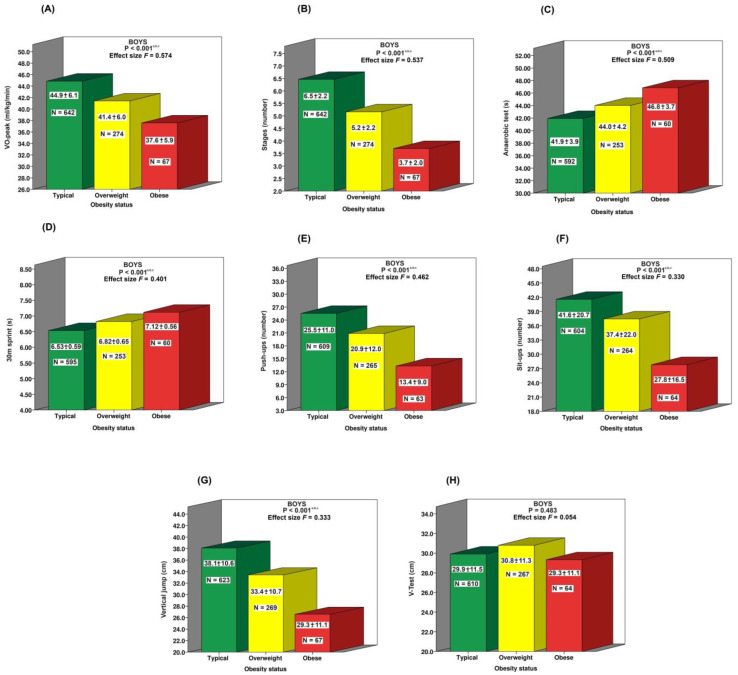
Comparison of physical performance of boys based on BMI status (typical, overweight, obese) on various physical fitness factors: (**A**) VO_2_peak;. (**B**) Stages; (**C**) Anaerobic test; (**D**) 30m sprint; (**E**) Push-ups; (**F**) Sit-ups; (**G**) Vertical jump; (**H**) V-test. Statistical significance was assessed using ANOVA comparisons. The *p*-values indicate the significance of differences between groups: “a” for typical vs. overweight; “b” for typical vs. obese; “c” for overweight vs. obese. Effect size *F*: 0.1 = small, 0.25 = moderate, 0.40 = large. The significance threshold was set at *p* < 0.05.

Figure 2 demonstrates that, overall, typical-BMI boys outperformed their overweight and obese counterparts in most fitness tests. This trend was also observed between overweight and obese teenagers. An exception to this tendency was flexibility (V-test), where BMI status appears to have had no significant impact.

**Figure 3:**
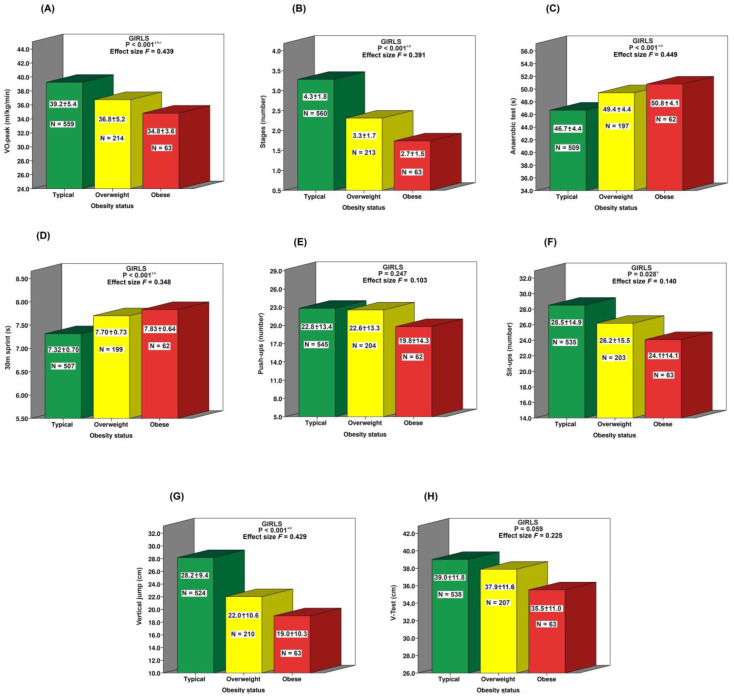
Comparison of physical performance of girls based on BMI status (typical, overweight, obese) on various physical fitness factors: (**A**) VO2peak;. (**B**) Stages; (**C**) Anaerobic test; (**D**) 30m sprint; (**E**) Push-ups; (**F**) Sit-ups; (**G**) Vertical jump; (**H**) V-test. Statistical significance was assessed using ANOVA comparisons. The p-values indicate the significance of differences between groups: “a” for typical vs. overweight; “b” for typical vs. obese; “c” for overweight vs. obese. Effect size F: 0.1 = small, 0.25 = moderate, 0.40 = large. The significance threshold was set at *p* < 0.05.

In contrast to boys, Figure 3 shows a lack of significant differences between the overweight and obese groups in most fitness assessment tests for girls. However, teenager girls with a typical BMI demonstrated superior physical performance compared to their overweight or obese peers across most physical tests, though this difference was less pronounced than in boys. Notably, performance on the V-test and push-ups appeared unaffected by BMI status among these girls.

Table 6 presents a comprehensive overview of body dissatisfaction among adolescents, highlighting its association with various factors such as gender, BMI, and socioeconomic status. The findings indicated a generally low satisfaction rate, with only 40% of boys and 35% of girls expressing satisfaction with their body image. A notable gender disparity was evident, as a greater proportion of girls (52.2%) desired thinness compared to boys (25.1%). Among those who were dissatisfied, over 80% of girls wished to be thinner, whereas only 41.8% of boys shared this sentiment.

Socioeconomic status significantly influenced body dissatisfaction in girls (*p* = 0.002) but did not appear to affect boys (*p* = 0.351). Lastly, self-assessment of body image demonstrated reliability, showing strong and significant correlations with independent evaluations (R = 0.767 and Kendall’s Tau-B = 0.969 for boys; R = 0.813 and Kendall’s Tau-B = 0.725 for girls).

Table 7 presents a comparison of anthropometric and physical fitness profiles between adolescent boys and girls with satisfied and dissatisfied body images. Both sexes with dissatisfied body image exhibited significantly higher anthropometric values, including BM, BMI, WHtR, and WC. Physical fitness scores, as assessed by the various tests in this study, were generally better among adolescents satisfied with their body image. A closer examination of individual test scores suggests that girls may be more susceptible to the effects of body dissatisfaction on physical fitness, with larger effect sizes (Cohen’s d) often exceeding 0.40. However, when combining the results of all fitness tests into a composite score, the effect sizes between sexes became comparable, with a global score around 0.40, highlighting the significant clinical importance of body dissatisfaction on overall physical fitness.

Table 8 presents a comparison of anthropometric profiles and physical performance between adolescents with typical BMI who are satisfied and dissatisfied with their body image. No significant differences were observed in age, BM, BH, BMI, WC, or WHtR between the two groups. These results suggest that, despite differences in body satisfaction, the groups shared similar body compositions, indicating that the anthropometric profile alone does not fully explain the level of dissatisfaction. Furthermore, for most physical performance variables, adolescents satisfied with their body image achieved significantly better results. These differences, although moderate in effect, reveal a clear association between body satisfaction and physical fitness performance.

## 4. Discussion

This study, conducted with a representative sample of 1862 Canadian adolescents from Québec, offers new and innovative insights into various physical and psychological characteristics of this population. Primarily, it enabled the update of normative values for fitness assessment tests commonly used in Canadian surveys, such as VO_2_peak, vertical jump, and sit-and-reach flexibility [18,19]. While these studies were rigorously conducted, it is important to note that the testing context differed considerably (home-based assessments), the participant age range was much broader (from 6 to 69 years), and the protocol used included a limited number of tests. Furthermore, the complex interactions among physical fitness performance, weight status as determined by BMI, and body image in Canadian adolescents remain poorly understood and warrant further exploration.

### 4.1. Standardization of Reference Values for Physical Fitness Tests in Adolescence

As previously stated, recent and diverse normative values for Canadian adolescents are relatively scarce. The two most recent Canadian surveys focused exclusively on four fitness tests: grip strength, VO_2_max, vertical jump, and the V-test for flexibility assessment [18,19]. This approach is explained by the methodological and logistical constraints faced by the authors. In the present study, targeting only students aged 12 to 17, collaboration with the school network facilitated easy access to a large participant sample and enabled greater diversity in the number and types of physical fitness tests administered. Consequently, additional physical fitness tests were incorporated to provide a more comprehensive profile of adolescents’ physical capacities.

With this goal in mind, two additional muscular endurance tests were included alongside the standard tests (VO_2_peak, countermovement jump, and sit-and-reach), namely, push-ups to assess upper body endurance and sit-ups for abdominal muscle endurance. This fundamental physical fitness marker had to be part of the battery of tests given its importance, particularly in adolescence [44]. However, rather than using the traditional method of the maximum number of repetitions in one minute, a protocol with an imposed rhythm was preferred. This approach, more rigorous on a methodological level, allowed us to homogenize experimental conditions by controlling the quality of each repetition in addition to the speed of execution. This last point supports the maintenance of a more regular submaximal execution speed, which emphasizes endurance over speed [39]. Given the challenges encountered during preliminary testing, the protocol was adjusted by substituting full sit-ups with half sit-ups. Previous studies with active Canadian adolescents demonstrated the effectiveness of full sit-ups with an imposed rhythm [45,46]. However, our preliminary data challenge the suitability of this procedure. Many adolescents encounter technical difficulties, making it challenging to perform a sufficient number of repetitions with proper form. This difficulty is another sign of a general decline in physical fitness over time, impacting both endurance and strength. This observation is consistent with global trends that have observed a substantial decrease in muscular endurance since 2010, a phenomenon that can probably be attributed to the rising prevalence of sedentary lifestyles among adolescents [44].

Finally, a test measuring anaerobic capacity was also introduced. While anaerobic power is a commonly assessed physiological factor in young athletes [47,48], it is rarely measured in general adolescent populations. However, a review of the different physical activities practiced during adolescence often involves the use of this energy source. This fundamental capacity is essential for a variety of physical activities, ranging from structured sports like soccer and hockey to recreational activities like dodgeball, kick-ball, or kin-ball. A deficit in anaerobic power can limit participation in these activities, even in non-competitive settings, potentially leading to increased sedentary behaviors.

This study achieved its primary objective of updating reference standards for physical fitness assessment tests in adolescents, based on age and gender. Additionally, the revision of several tests and the inclusion of new assessment procedures provide a more comprehensive analysis of physical fitness by incorporating measures that cover all key physical fitness determinants. While these standards are specifically established for the Québec population, they can serve as a valuable benchmark for comparison both in Canada and internationally.

### 4.2. Relationship Between Obesity Status and Physical Fitness Level Assessment

It is well established that overweight and obesity have a detrimental impact on the cardiorespiratory fitness of children and adolescents. A recent Canadian study quantified this impairment, revealing that overweight and obese boys and girls reach critical VO_2_peak thresholds at relatively young ages [10]. Specifically, boys exhibit VO_2_peak values below 42 mL/kg/min from the age of 11, while girls reach a threshold of 35 mL/kg/min from the age of 14. These thresholds lower than the reference values are associated with an elevated risk of early-onset cardiometabolic diseases [49]. However, this type of information is less available for muscle or joint markers [50], particularly within the Canadian population. Regarding the other physical fitness tests, it is clear that overweight and obesity significantly impair performance, particularly in boys. Furthermore, the deterioration in performance is exacerbated when overweight progresses to obesity. In contrast, girls exhibit a less pronounced impact, with only VO_2_peak being significantly affected by the transition from overweight to obesity. Surprisingly, there were no significant differences between overweight and obese girls across most fitness markers. This homogeneity can be partly attributed to the generally lower baseline physical fitness of overweight or obese girls compared to boys. Furthermore, the earlier cessation of physical activity among girls in favor of sedentary pursuits exacerbates this disparity, even among those with a normal BMI. This phenomenon contributes to a widening gender gap in physical activity levels and underscores the crucial need to encourage girls to adopt and maintain an active lifestyle. This initial disparity could mask the additional differences induced by overweight or obesity, making it challenging to identify significant variations within the female group. This hypothesis is especially relevant for short-duration activities or those requiring partial body mass support, such as modified push-ups. For this specific marker, no significant differences were found among girls across the three BMI statuses, unlike the results observed in boys. Joint mobility, as measured by the V-test, did not appear to be influenced by obesity status in either sex.

Thus, identifying all factors that may contribute to these results is a complex task. For VO_2_peak, which is also the most impacted variable in both sexes, it is reasonable to assume that the decrease in performance is directly related to excess fat mass (dead load), which requires additional muscular and cardiorespiratory effort during prolonged exercise. While excess body fat is a significant factor contributing to decreased physical performance across multiple markers, other factors likely play a role. A recent study examining the decline in VO_2_peak among children and youth over the past 35 years revealed that performance differences persisted even after adjusting for obesity status [10]. Similarly, a meta-analysis involving over 9 million children and adolescents identified a decrease in muscular fitness that cannot be fully attributed to obesity alone [44]. These two studies converge on a common observation, namely, that the increase in sedentary behaviors appears to be the primary driver of this overall decline in physical fitness level, an observation also reported elsewhere [29]. However, while the decline in overall physical fitness level among adolescents is worrying, it is important to note that this trend is reversible. By implementing effective physical activity programs specifically designed for overweight or obese youth, significant improvements in physical fitness level can be achieved [50], which in turn can greatly reduce their long-term risk of developing serious cardiometabolic diseases.

### 4.3. Impact of Physical Fitness Level on Body Image Dissatisfaction

The present study on body image among Canadian adolescents from Québec revealed significant trends. Firstly, body dissatisfaction was prevalent, affecting 60% of boys and 65% of girls. These figures, consistent with existing research, underscore the importance of this issue among boys, even though girls are more likely to desire thinness [23,51]. The disparity in body aspirations between sexes is pronounced: over 80% of girls aimed to be thinner, while only 41.8% of boys shared this desire. This aligns with the literature suggesting that girls prioritize thinness, while boys aspire to gain muscle mass [52]. Secondly, a substantial proportion of adolescents (40% of boys and 35% of girls) expressed satisfaction with their body image. This finding is noteworthy, as our sample demonstrated a valid self-assessment of body image. The strong correlations between self-assessments and independent evaluations (R = 0.767 and Kendall’s Tau-B = 0.969 for boys; R = 0.813 and Kendall’s Tau-B = 0.725 for girls) confirm the reliability of adolescent self-reports. However, despite this objective self-assessment, nearly two-thirds of the adolescents exhibited body dissatisfaction. This underscores the deeply internalized nature of body dissatisfaction, influenced by often unattainable societal ideals [53].

The present findings indicated an exceptionally high prevalence of body dissatisfaction among obese adolescents, with 85% of boys and 90% of girls reporting such dissatisfaction. This correlation raises an essential question: is obesity the cause or the consequence of body dissatisfaction? Some experts propose that eating disorders, often observed in overweight adolescents, may play a significant role. These disruptive behaviors, such as fasting or skipping meals, can paradoxically contribute to weight gain. While our study does not fully resolve this question, it demonstrates that body dissatisfaction is not confined to overweight or obese adolescents. Nearly half of those with a typical BMI also reported dissatisfaction with their bodies. This underscores the importance of recognizing body dissatisfaction as a critical issue extending beyond obesity. Thus, health professionals should prioritize body dissatisfaction, regardless of an adolescent’s BMI, as it is a major risk factor for the development of severe eating disorders like anorexia nervosa [54].

While a high level of physical fitness brings clear physical health benefits, it may also have important effects on some psychosocial factors such as body image. This study illustrates that adolescents who feel dissatisfied with their body image tend to perform worse on most physical tests than those who are satisfied with their appearance. At first glance, this finding may seem predictable, as adolescents who feel dissatisfied are also often overweight or obese [25,26,27] and carrying excess fat mass can directly impact physical performance, limiting their ability to excel in fitness-related tasks. Our results are also consistent with the fact that body dissatisfaction is linked to a decrease in physical activity during adolescence [55]. However, our results reveal a more nuanced picture. Indeed, body dissatisfaction was not limited to overweight or obese adolescents. A substantial portion of adolescents in this study with a typical BMI (~50%) also experienced body dissatisfaction. When comparing adolescents within this BMI range, we observed significantly better physical performance among those who felt satisfied with their body image. This finding suggests that physical fitness plays a role in shaping body satisfaction as a psychological factor, independently of weight-related challenges. In other words, fitness level may enhance body satisfaction even for adolescents who fall within a normal BMI range, highlighting the importance of fitness in fostering a positive body image. Indeed, the scientific literature extensively demonstrated a strong link between physical activity and a positive body image, defined by appreciation, acceptance, and self-respect [28,29,30]. This connection appears to be further reinforced by the physical benefits derived from regular exercise. Our findings align with this evidence: adolescents who are satisfied with their body image consistently exhibited significantly higher levels of physical fitness across all assessed variables. Furthermore, the connection between fitness and body image is not just about appearance; it is deeply tied to feelings of strength, capability, and overall well-being.

Adolescents who engage in regular physical activity reported higher self-esteem and a greater sense of accomplishment, which may contribute to their positive body image and improved mental health. Substantially reducing sedentary behavior is crucial for improving physical fitness. Engaging in physical activity or sports not only benefits physical health but also contributes to overall psychosocial well-being. Among these factors, body image plays a pivotal role, particularly in influencing the psychological well-being of adolescents [24,56]. As many studies report, a good level of physical fitness contributes significantly to the improvement of body image satisfaction [28,29,30,57]. This improvement in body image satisfaction has been linked to greater self-esteem, confidence, and social acceptance, all of which are critical for positive mental health. Thus, by encouraging adolescents to adopt a more physically active lifestyle, they will not only experience improved physical health but also enhanced psychological well-being due to greater satisfaction with their body image. These benefits, including better mental health and a more positive self-perception, extend into adulthood [58]. As such, promoting physical activity early on can lead to long-term improvements in both physical and psychological health, fostering a generation that is both healthier and more confident in their bodies.

### 4.4. Body Image Dissatisfaction vs. Socioeconomic Status

The Québec Ministry of Education has developed a socioeconomic deprivation index specifically tailored for Québec schools [59]. This index, assigned to each school, reflects the proportion of families, with at least one child, whose income falls near or below the low-income threshold. The index scale ranges from 1 to 10, with 1 representing the highest socioeconomic status. For this study, schools with scores from 1 to 5 were considered to have a high socioeconomic status, while those with scores from 6 to 10 were classified as having a low socioeconomic status. Based on this classification and as reported earlier, our results revealed a significant association between socioeconomic status and body dissatisfaction in girls (*p* = 0.002), but not in boys (*p* = 0.351), which is consistent with results reported elsewhere [60,61]. It is clearly established that lower socioeconomic status is a risk factor for overweight/obesity. One explication is that people with low income often eat less fresh fruit and vegetables and more processed foods. For the difference between girls and boys, it could be explained by sociocultural and psychological factors [30,56,60]. It can be hypothesized that adolescent girls from higher socioeconomic backgrounds often have greater access to products and services focused on appearance management, such as healthy foods, which may help them conform to idealized body standards and, consequently, reduce their dissatisfaction. Such considerations appear to resonate less with boys, who seem less susceptible to these sociocultural influences. Nonetheless, further investigation into this matter is warranted.

### 4.5. Limitations and Strength

This study, while informative, presents certain limitations. Its cross-sectional design precludes the establishment of causal relationships. Although the sample is representative of Québec adolescents, generalization to other geographic contexts should be approached with caution, especially given the absence of data from rural areas. Nonetheless, the study possesses several strengths. The substantial sample size (N = 1862) ensures robust representativeness of Québec adolescents. The anthropometric measurements and physical fitness tests employed in this study are well-established and reliable tools. The assessment of body dissatisfaction, while a widely used method in the literature, presents certain limitations. The inclusion of an external silhouette evaluator strengthens the validity of our results. Furthermore, to our knowledge, this is one of the first Canadian studies to delve deeply into the associations between physical fitness, particularly muscular components, and obesity, as well as maximal anaerobic power, in a large adolescent sample. Finally, it provides a novel perspective on the relationship between body dissatisfaction and physical fitness level.

## 5. Conclusions

In light of the three initial objectives outlined, one can confidently state that they have been achieved. First, this study has provided an objective assessment of the physical fitness of French-Canadian adolescents and established new standards for several parameters, including aerobic and anaerobic capacity, muscular endurance, muscular power, and joint mobility. Second, it has highlighted a significant association between obesity and poorer physical performance. Finally, our results strongly suggest that higher levels of physical fitness are linked to greater body satisfaction, even among adolescents of typical BMI. This finding is of paramount importance and underscores the need for all professionals working with adolescents to prioritize their body image concerns. Our findings reveal that a substantial proportion of adolescents, even those within the ’typical’ BMI range, express dissatisfaction with their appearance. This dissatisfaction can have significant detrimental consequences on their physical, psychological, and social well-being. Therefore, improving physical fitness not only enhances physical health but also contributes to psychological well-being. However, longitudinal studies are necessary to further explore the causal relationships among these variables.

## Figures and Tables

**Table 1:** Age-related anthropometric and physical fitness profiles of Canadian adolescents from Québec.

Boys						
Variables	12 Years N = 122	13 Years N = 223	14 Years N = 165	15 Years N = 200	16 Years N = 154	17 Years N = 86
BM (kg)	48.2 ± 9.9	54.8 ± 13.1	57.6 ± 10.2	62.9 ± 11.1	66.4 ± 11.7	68.9 ± 11.6
BH (cm)	155.1 ± 8.6	161.3 ± 9.0	166.5 ± 7.7	170.6 ± 6.6	172.7 ± 7.6	173.5 ± 7.8
BMI (kg/m^2^)	20.7 ± 4.2	20.9 ± 4.0	21.2 ± 3.9	21.9 ± 4.1	22.5 ± 4.4	23.5 ± 4.4
WC (cm)	73.2 ± 10.6	74.8 ± 10.3	76.4 ± 10.6	78.3 ± 10.3	80.2 ± 10.2	82.3 ± 10.1
WHtR	0.47 ± 0.06	0.46 ± 0.06	0.46 ± 0.06	0.46 ± 0.06	0.46 ± 0.06	0.47 ± 0.07
VO_2_peak	44.8 ± 5.3	43.8 ± 4.9	44.5 ± 6.7	43.0 ± 6.9	42.8 ± 7.3	40.9 ± 7.3
Stages (nb)	5.1 ± 2.0	5.3 ± 1.9	6.1 ± 2.5	6.2 ± 2.5	6.6 ± 2.5	6.6 ± 2.4
Anaerobic (s)	44.7 ± 4.1	44.7 ± 4.3	42.4 ± 4.1	41.8 ± 4.0	41.5 ± 3.5	41.1 ± 3.7
Sprint (s)	6.94 ± 0.62	6.96 ± 0.64	6.57 ± 0.58	6.49 ± 0.59	6.46 ± 0.53	6.33 ± 0.47
Push-ups (nb)	20.1 ± 11.5	19.4 ± 10.9	23.2 ± 10.8	25.4 ± 11.5	26.7 ± 12.1	27.9 ± 10.3
Sit-ups (nb)	41.2 ± 21.3	36.7 ± 21.0	37.6 ± 17.4	41.0 ± 22.0	41.2 ± 23.0	40.7 ± 20.2
Jump (cm)	28.6 ± 10.1	31.4 ± 8.6	36.4 ± 10.4	38.0 ± 9.0	42.3 ± 10.7	41.3 ± 12.6
V-test (cm)	30.5 ± 10.1	27.3 ± 10.9	31.0 ± 11.4	31.7 ± 11.1	31.3 ± 11.6	29.2 ± 13.0
**Girls**						
**Variables**	**12 years ** **N = 151**	**13 years** **N = 205**	**14 years** **N = 134**	**15 years** **N = 112**	**16 years** **N = 126**	**17 years** **N = 66**
BM (kg)	47.9 ± 9.4	51.6 ± 10.6	55.3 ± 10.1	56.9 ± 8.7	58.8 ± 9.2	59.7 ± 9.9
BH (cm)	154.9 ± 6.3	157.5 ± 6.1	158.3 ± 6.2	161.5 ± 6.7	160.0 ± 7.0	162.7 ± 7.8
BMI (kg/m^2^)	20.8 ± 4.3	21.1 ± 4.1	23.0 ± 4.8	22.1 ± 3.4	22.8 ± 4.1	23.0 ± 4.1
WC (cm)	73.5 ± 10.1	74.5 ± 10.1	75.7 ± 9.8	76.6 ± 10.1	77.7 ± 10.0	78.6 ± 10.0
WHtR	0.48 ± 0.06	0.47 ± 0.06	0.48 ± 0.06	0.47 ± 0.06	0.48 ± 0.05	0.49 ± 0.06
VO_2_peak	41.3 ± 4.3	39.5 ± 4.6	37.3 ± 4.3	38.3 ± 5.6	36.0 ± 6.0	33.8 ± 5.5
Stages (nb)	3.8 ± 1.7	3.7 ± 1.7	3.5 ± 1.6	4.5 ± 2.0	4.3 ± 2.1	4.1 ± 1.8
Anaerobic (s)	49.0 ± 4.2	47.9 ± 4.2	47.7 ± 4.7	46.9 ± 4.6	47.2 ± 5.2	46.7 ± 4.8
Sprint (s)	7.66 ± 0.64	7.50 ± 0.69	7.43 ± 0.75	7.32 ± 0.72	7.40 ± 0.80	7.25 ± 0.78
Push-ups (nb)	24.2 ± 14.1	22.1 ± 13.2	22.2 ± 13.0	22.9 ± 14.6	22.4 ± 13.0	19.1 ± 12.5
Sit-ups (nb)	28.9 ± 14.6	24.2 ± 11.8	25.5 ± 13.0	29.5 ± 16.7	31.9 ± 19.2	27.0 ± 15.1
Jump (cm)	20.6 ± 9.9	22.9 ± 9.6	24.1 ± 8.4	31.1 ± 9.0	30.3 ± 10.2	31.5 ± 9.5
V-test (cm)	38.3 ± 11.1	39.0 ± 12.0	38.6 ± 11.4	38.2 ± 11.9	38.7 ± 11.6	37.2 ± 12.2

N = Number of participants; Values are expressed as means ± standard deviations; VO_2_peak is expressed in ml/kg/min.

**Table 2:** Smoothed percentile standards for the VO_2_peak (mL/kg/min) and the number of 1 min stage completed (Nb) according to age and sex in Québec adolescents.

						Percentiles VO_2_peak (N = 1862)					
	N	L	M	S	3	10	25	50	75	90	97
Boys											
12 years	128	1.00	44.6	0.118	34.7	37.9	41.1	44.6	48.2	51.4	54.5
13 years	225	1.01	43.8	0.112	34.6	37.5	40.5	43.8	47.1	50.1	53.0
14 years	181	0.65	44.7	0.151	32.7	36.3	40.2	44.7	49.3	53.6	58.0
15 years	215	0.99	43.1	0.161	30.1	34.2	38.4	43.1	47.8	52.0	56.2
16 years	163	1.00	42.6	0.171	28.9	33.3	37.7	42.6	47.5	51.9	56.3
17 years	96	1.01	41.5	0.179	27.5	32.0	36.5	41.5	46.5	51.0	55.5
Girls											
12 years	166	−0.41	41.3	0.104	34.2	36.3	38.5	41.3	44.3	47.4	50.6
13 years	214	1.21	39.2	0.117	32.3	34.2	36.4	39.2	42.6	46.3	50.6
14 years	145	−0.30	37.0	0.115	30.0	32.0	34.3	37.0	40.0	43.0	46.3
15 years	124	0.24	37.9	0.146	28.5	31.3	34.3	37.9	41.8	45.5	49.5
16 years	133	−0.51	36.6	0.167	27.4	29.9	32.8	36.6	41.1	45.9	51.5
17 years	72	−0.10	34.1	0.163	25.2	27.7	30.6	34.1	38.1	42.1	46.6
						**Percentiles 1 min stages (N = 1862)**					
	**N**	**L**	**M**	**S**	**3**	**10**	**25**	**50**	**75**	**90**	**97**
Boys											
12 years	128	1.00	5.0	0.392	1.3	2.5	3.7	5.0	6.3	7.5	8.7
13 years	225	0.99	5.5	0.359	1.8	3.0	4.2	5.5	6.8	8.0	9.2
14 years	181	0.71	6.2	0.410	2.0	3.2	4.6	6.2	8.0	9.7	11.5
15 years	215	0.86	6.1	0.403	1.8	3.1	4.6	6.1	7.9	9.5	11.1
16 years	163	1.00	6.5	0.379	1.8	3.3	4.8	6.5	8.2	9.7	11.1
17 years	96	1.01	6.5	0.364	2.0	3.5	4.9	6.5	8.1	9.5	11.0
Girls											
12 years	166	0.58	3.7	0.460	1.1	1.8	2.6	3.7	4.9	6.1	7.5
13 years	214	1.00	3.8	0.460	0.5	1.6	2.6	3.8	5.0	6.0	7.1
14 years	145	0.64	3.1	0.457	0.9	1.5	2.2	3.1	4.1	5.1	6.2
15 years	124	0.62	4.2	0.444	1.3	2.1	3,0	4.2	5.5	6.8	8.2
16 years	133	0.51	4.3	0.488	1.2	2.0	3.0	4.3	5.8	7.4	9.1
17 years	72	1.00	4.0	0.475	0.4	1.6	2.7	4.0	5.3	6.4	7.6

N = number of participants; L = Lambda; M = median; S = coefficient of variation.

**Table 3:** Smoothed percentile standards for the 30 m sprint (s) and the anaerobic test (s) according to age and sex in Québec adolescents.

						Percentiles 30 m sprint (N = 1685)					
	N	L	M	S	3	10	25	50	75	90	97
Boys											
12 years	116	1.00	6.85	0.089	8.00	7.63	7.26	6.85	6.44	6.07	5.70
13 years	210	−1.38	6.97	0.092	8.49	7.92	7.43	6.96	6.56	6.24	5.96
14 years	163	−1.12	6.57	0.088	7.89	7.42	6.99	6.57	6.21	5.91	5.65
15 years	191	−2.33	6.47	0.091	8.05	7.42	6.92	6.47	6.11	5.84	5.61
16 years	149	−1.79	6.44	0.082	7.72	7.24	6.83	6.44	6.11	5.85	5.62
17 years	84	1.00	6.30	0.074	7.18	6.90	6.62	6.30	5.99	5.70	5.43
Girls											
12 years	166	151	1.00	7.61	0.083	8.81	8.43	8.05	7.62	7.20	6.81
13 years	214	200	−0.82	7.45	0.091	8.96	8.42	7.94	7.45	7.02	6.67
14 years	145	133	0.99	7.30	0.101	8.69	8.25	7.80	7.30	6.80	6.36
15 years	124	108	1.01	7.23	0.098	8.56	8.14	7.71	7.23	6.75	6.32
16 years	133	115	1.00	7.34	0.109	8.84	8.36	7.88	7.34	6.80	6.31
17 years	72	65	−0.40	7.14	0.107	8.80	8.22	7.68	7.14	6.65	6.24
						**Percentiles anaerobic test (N = 1683)**					
	**N**	**L**	**M**	**S**	**3**	**10**	**25**	**50**	**75**	**90**	**97**
Boys											
12 years	116	1.00	44.33	0.092	52.00	49.56	47.08	44.33	41.58	39.10	36.66
13 years	207	−1.25	44.75	0.097	55.05	51.23	47.91	44.75	42.02	39.87	37.97
14 years	164	−1.29	42.53	0.096	52.23	48.63	45.50	42.53	39.97	37.94	36.16
15 years	188	−2.12	41.79	0.095	52.32	48.12	44.77	41.79	39.35	37.50	35.91
16 years	149	−1.58	41.56	0.084	49.85	46.77	44.10	41.56	39.37	37.63	36.09
17 years	87	−2.20	41.00	0.090	50.68	46.84	43.76	41.00	38.73	36.99	35.50
Girls											
12 years	152	1.00	48.76	0.085	56.56	54.07	51.55	48.76	45.97	43.45	40.96
13 years	201	−0.80	47.79	0.088	57.08	53.79	50.78	47.79	45.10	42.90	40.91
14 years	133	0.99	47.11	0.098	55.80	53.03	50.22	47.11	44.00	41.20	38.44
15 years	107	−2.52	46.61	0.097	59.52	54.11	50.06	46.61	43.87	41.83	40.11
16 years	114	1.00	46.64	0.111	56.38	53.28	50.13	46.64	43.15	40.00	36.90
17 years	65	1.01	46.04	0.106	55.21	52.29	49.33	46.04	42.75	39.78	36.85

N = number of participants; L = Lambda; M = median; S = coefficient of variation.

**Table 4:** Smoothed percentile standards for the cadence push-ups (Nb) and the cadence sit-ups (Nb) according to age and sex in Québec adolescents.

				Percentiles cadence push-ups (N = 1778)							
	N	L	M	S	3	10	25	50	75	90	97
Boys											
12 years	122	0.38	21.3	0.572	5.3	9.0	14.0	21.3	30.5	40.7	52.5
13 years	211	0.50	19.8	0.573	4.2	7.9	12.9	19.8	28.2	37.0	46.9
14 years	173	1.00	23.0	0.466	2.8	9.3	15.8	23.0	30.2	36.7	43.2
15 years	212	1.00	25.0	0.453	3.7	10.5	17.4	25.0	32.6	39.5	46.3
16 years	157	0.99	26.0	0.464	3.3	10.5	17.9	26.0	34.1	41.5	48.7
17 years	84	1.01	30.0	0.373	9.0	15.7	22.5	30.0	37.5	44.3	51.1
Girls											
12 years	158	0.52	23.3	0.630	4.1	8.3	14.1	23.3	32.7	43.8	56.4
13 years	211	0.48	22.4	0.618	4.9	9.2	15.1	22.4	33.5	44.3	56.6
14 years	142	0.47	23.3	0.586	3.8	8.3	14.6	23.3	35.0	47.0	60.7
15 years	119	0.50	23.7	0.638	4.6	9.0	14.9	23.7	33.0	43.3	54.8
16 years	123	0.52	23.1	0.580	3.6	7.0	12.0	23.1	29.3	40.2	53.1
17 years	66	0.40	19.5	0.655	5.3	9.0	14.0	19.5	30.5	40.7	52.5
				**Percentiles cadence sit-ups (N = 1740)**							
	**N**	**L**	**M**	**S**	**3**	**10**	**25**	**50**	**75**	**90**	**97**
Boys											
12 years	122	−0.10	38.0	0.517	15.0	20.0	27.0	38.0	54.2	75.4	100.0
13 years	214	0.37	34.8	0.572	8.8	14.8	23.0	34.8	49.9	66.6	86.1
14 years	172	0.24	38.1	0.463	14.3	20.1	27.5	38.1	51.5	66.4	84.0
15 years	208	0.48	40.0	0.537	8.6	15.7	25.7	40.0	58.1	77.7	100.0
16 years	152	−0.03	40.8	0.558	14.5	20.1	28.1	40.8	59.6	84.1	100.0
17 years	87	0.07	41.4	0.496	15.8	21.6	29.5	41.4	57.6	77.1	100.0
Girls											
12 years	158	0.29	26.0	0.512	8.4	12.6	18.1	26.0	36.1	47.4	60.8
13 years	206	0.66	24.1	0.488	5.9	10.8	16.6	24.1	32.5	40.7	49.4
14 years	141	0.62	25.5	0.510	5.9	11.0	17.3	25.5	34.8	44.1	54.1
15 years	113	0.36	29.2	0.566	7.6	12.6	19.4	29.2	41.7	55.6	71.9
16 years	128	0.18	32.3	0.602	9.1	14.1	21.2	32.3	47.8	66.5	90.5
17 years	69	0.20	28.2	0.560	8.6	13.0	19.0	28.2	40.6	55.2	73.3

N = number of participants; L = Lambda; M = median; S = coefficient of variation.

**Table 5:** Smoothed percentile standards for the vertical jump (cm) and the V-test (cm) according to age and sex in Québec adolescents.

						Percentiles vertical jump (N = 1778)					
	N	L	M	S	3	10	25	50	75	90	97
Boys											
12 years	124	1.00	28.9	0.353	9.7	15.8	22.0	28.9	35.8	42.0	48.1
13 years	218	0.99	31.0	0.274	15.1	20.1	25.3	31.0	36.7	41.9	47.0
14 years	175	1.01	36.7	0.286	16.9	23.2	29.6	36.7	43.8	50.1	56.4
15 years	206	1.00	38.6	0.237	21.4	26.9	32.4	38.6	44.8	50.3	55.8
16 years	156	1.00	42.3	0.253	22.2	28.6	35.1	42.3	49.5	56.0	62.4
17 years	92	1.01	41.6	0.305	17.6	25.3	33.0	41.6	50.1	57.8	65.4
Girls											
12 years	151	1.00	23.8	0.419	5.0	11.0	17.1	23.8	30.5	36.6	42.6
13 years	199	1.00	25.3	0.349	8.7	14.0	19.3	25.3	31.3	36.6	41.9
14 years	139	0.40	29.9	0.289	16.2	20.0	24.4	29.9	36.1	42.2	48.9
15 years	119	0.14	30.3	0.337	15.6	19.4	24.1	30.3	37.9	46.1	55.6
16 years	129	0.99	31.4	0.302	13.6	19.2	25.0	31.4	37.8	43.6	49.2
17 years	70	1.00	28.9	0.353	9.7	15.8	22.0	28.9	35.8	42.0	48.1
						**Percentiles V-test (N = 1772)**					
	**N**	**L**	**M**	**S**	**3**	**10**	**25**	**50**	**75**	**90**	**97**
Boys											
12 years	116	1.00	31.0	0.331	11.7	17.8	24.1	31.0	37.9	44.2	50.3
13 years	214	1.00	27.0	0.399	6.7	13.2	19.7	27.0	34.3	40.8	47.3
14 years	174	0.99	30.5	0.368	9.5	16.2	22.9	30.5	38.1	44.9	51.7
15 years	205	1.01	33.0	0.350	11.2	18.2	25.2	33.0	40.8	47.8	54.7
16 years	154	1.01	33.0	0.372	9.8	17.2	24.7	33.0	41.3	48.7	56.0
17 years	93	0.99	30.0	0.445	5.0	12.9	21.0	30.0	39.0	47.2	55.2
Girls											
12 years	156	1.00	38.0	0.290	17.3	23.9	30.6	38.0	45.4	52.1	58.7
13 years	210	1.00	40.3	0.308	17.0	24.4	31.9	40.3	48.7	56.2	63.6
14 years	142	1.01	38.0	0.295	16.8	23.6	30.4	38.0	45.5	52.3	59.0
15 years	114	1.13	39.3	0.312	15.0	23.1	30.9	39.3	47.5	54.7	61.6
16 years	125	0.99	39.0	0.300	17.1	24.0	31.1	39.0	46.9	54.0	61.1
17 years	69	1.00	38.0	0.328	14.6	22.0	29.6	38.0	46.4	54.0	61.4

N = number of participants; L = Lambda; M = median; S = coefficient of variation.

**Table 6:** Global prevalence of body image dissatisfaction and its associations with BMI, socioeconomic status, and validation of body image self-assessment tool.

Overall Body Image Dissatisfaction				
All Participants	Boys		Girls	
	N = 885	%	N = 803	%
Satisfied	354	40.0	281	35.0
Want to be thinner	222	25.1	419	52.2
Want to be bigger	309	34.9	103	12.8
Body image dissatisfaction based on the desire to be thinner or bigger				
Dissatisfied	N = 531	%	N = 522	%
Want to be thinner	222	41.8	419	80.3
Want to be bigger	309	58.2	103	19.7
Body image dissatisfaction vs. BMI				
Typical BMI	N = 569	%	N = 533	%
Satisfied	274	48.2	239	44.8
Dissatisfied	295	51.8	294	55.2
Overweight	N = 245	%	N = 200	%
Satisfied	68	27.8	34	17.0
Dissatisfied	177	72.2	166	83.0
Obese	N = 61	%	N = 60	%
Satisfied	9	14.8	6	10.0
Dissatisfied	52	85.2	54	90.0
Body image dissatisfaction vs. socioeconomic status				
	Mean	SD	Mean	SD
Favorable	1.58	0.49	1.59	0.49
Unfavorable	1.61	0.49	1.69	0.46
*p* values (Student’s t-test)	0.351		0.002	
Validation of self-assessment of body image				
	R (N = 763)	Kendall’s Tau-B	R (N = 689)	Kendall’s Tau-B
Independent rating	0.767	0.696	0.813	0.725

BMI = body mass index; N = number of participants; R = Spearman’s correlation coefficient; % = percentage; SD = standard deviation; *p* values = significant at *p* ≤ 0.05.

**Table 7:** Sex-related comparisons of anthropometric and physical fitness profiles in adolescents with satisfied versus dissatisfied body images.

Boys						
	Satisfied	N	Dissatisfied	N	*p* Values	Cohen’s d
Age	14.9 ± 1.5	354	14.9 ± 1.6	531	0.640	0.00
BM (kg)	57.0 ± 11.2	338	61.2 ± 13.9	505	**<0.001**	0.33
BH (cm)	166.2 ± 10.1	352	166.6 ± 10.2	522	0.503	0.00
BMI (kg/m^2^)	20.9 ± 3.4	350	22.3 ± 4.5	525	**<0.001**	0.34
WC (cm)	74.6 ± 9.3	343	78.8 ± 11.3	520	**<0.001**	0.40
WHtR	0.45 ± 0.05	344	0.47 ± 0.07	522	**<0.001**	0.32
VO_2_peak (mL/kg/min)	44.5 ± 6.6	354	42.7 ± 6.4	531	**<0.001**	0.28
Stages (nb)	6.3 ± 2.3	354	5.7 ± 2.3	531	**<0.001**	0.26
Anaerobic power (s)	42.2 ± 3.8	333	43.2 ± 4.5	499	**0.001**	0.24
Sprint (s)	6.55 ± 0.56	333	6.71 ± 0.66	499	**<0.001**	0.26
Push-ups (nb)	25.8 ± 11.6	342	21.6 ± 11.3	518	**<0.001**	0.37
Sit-ups (nb)	41.9 ± 19.9	336	37.2 ± 21.3	517	**0.001**	0.23
Vertical jump (cm)	38.1 ± 10.4	341	34.7 ± 11.4	524	**<0.001**	0.31
V-test (cm)	30.9 ± 10.8	343	29.7 ± 11.7	517	0.136	0.11
Cumulative score	55.0 ± 17.6	297	47.7 ± 19.5	468	**<0.001**	0.39
**Girls**						
Age	14.7 ± 1.5	281	14.7 ± 1.6	522	0.070	0.00
BM (kg)	51.3 ± 9.1	273	55.5 ± 10.8	480	**<0.001**	0.41
BH (cm)	159.0 ± 7.2	275	158.6 ± 6.9	510	0.439	0.06
BMI (kg/m^2^)	20.4 ± 3.4	278	22.7 ± 4.5	516	**<0.001**	0.55
WC (cm)	72.2 ± 8.8	275	77.4 ± 10.3	514	**<0.001**	0.53
WHtR	0.46 ± 0.05	274	0.49 ± 0.06	514	**<0.001**	0.53
VO_2_peak (mL/kg/min)	39.4 ± 5.4	280	37.5 ± 5.2	522	**<0.001**	0.36
Stages (nb)	4.2 ± 1.8	281	3.7 ± 1.8	521	**<0.001**	0.28
Anaerobic power (s)	46.9 ± 4.6	256	48.3 ± 4.4	483	**<0.001**	0.31
Sprint (s)	7.33 ± 0.73	255	7.55 ± 0.70	484	**<0.001**	0.31
Push-ups (nb)	22.8 ± 14.2	278	22.5 ± 13.0	513	0.731	0.00
Sit-ups (nb)	29.0 ± 14.5	266	26.2 ± 15.0	508	**0.011**	0.19
Vertical jump (cm)	27.6 ± 9.8	257	24.5 ± 10.3	503	**<0.001**	0.31
V-test (cm)	39.6 ± 12.0	271	37.8 ± 11.5	509	**0.037**	0.15
Cumulative score	53.7 ± 18.2	225	46.5 ± 18.0	447	**<0.001**	0.40

N = number of participants; scores are presented as mean ± SD = standard deviation; *p* values bold = significant *p* ≤ 0.05; Cohen’s d = effect size: 0.20 = small effect; 0.50 = moderate effect; 0.80 = large effect; cumulative score (percentile) = Σ (VO_2_peak + stage + 30 m sprint + anaerobic test + push-ups + sit-ups + vertical jump + V-test)/8.

**Table 8:** Impact of body dissatisfaction on anthropometric profile and physical fitness performance in adolescents with typical BMI.

Variables	Satisfied	N	Dissatisfied	N	*p* Values	Cohen’s d
Age	14.7 ± 1.5	513	14.9 ± 1.6	589	0.066	0.13
BM (kg)	52.2 ± 9.6	504	51.7 ± 10.1	579	0.397	0.05
BH (cm)	162.8 ± 9.6	510	162.5 ± 9.9	581	0.551	0.03
BMI (kg/m^2^)	19.6 ± 2.6	511	19.4 ± 3.0	589	0.156	0.07
WC (cm)	71.4 ± 8.0	503	71.7 ± 8.2	587	0.548	0.04
WHtR	0.44 ± 0.04	503	0.44 ± 0.05	587	0.226	0.00
VO_2_peak (mL/kg/min)	42.8 ± 6.5	512	41.4 ± 6.3	589	**<0.001**	0.22
Stages (nb)	5.6 ± 2.3	513	5.2 ± 2.3	589	**0.005**	0.17
Anaerobic power (s)	44.0 ± 4.7	479	44.5 ± 4.9	549	**0.050**	0.10
Sprint (s)	6.86 ± 0.73	479	7.00 ± 0.77	548	**0.023**	0.19
Push-ups (nb)	24.8 ± 12.8	501	23.5 ± 11.8	582	0.083	0.11
Sit-ups (nb)	36.5 ± 18.9	486	33.5 ± 19.2	572	**0.013**	0.16
Vertical jump (cm)	34.2 ± 10.8	482	32.4 ± 11.7	569	**0.009**	0.16
V-test (cm)	34.8 ± 12.5	497	33.9 ± 12.6	570	0.244	0.07
Cumulative score	56.0 ± 17.1	421	52.8 ± 17.6	509	**0.005**	0.18

N = number of participants; scores are presented as mean ± SD = standard deviation; *p* values bold = significant *p* ≤ 0.05; Cohen’s d = effect size: 0.20 = small effect; 0.50 = moderate effect; 0.80 = large effect; cumulative score (percentile) = Σ (VO_2_peak + stage + 30 m sprint + anaerobic test + push-ups + sit-ups + vertical jump + V-test)/8.

## Data Availability

The original contributions presented in the study are included in the article; further inquiries can be directed to the corresponding author.
